# Supplementary material for: Microbiological and Cytokine Profiling of Menstrual Blood for the Assessment of Endometrial Receptivity: A Pilot Study
Source: Biomedicines. 2023 Apr 26;11(5):1284. doi: 10.3390/biomedicines11051284 (PMC10215904; doi:10.3390/biomedicines11051284)
Supplement: Supplementary file 1 [file biomedicines-11-01284-s001.zip › biomedicines-2288794-supplementary.pdf]

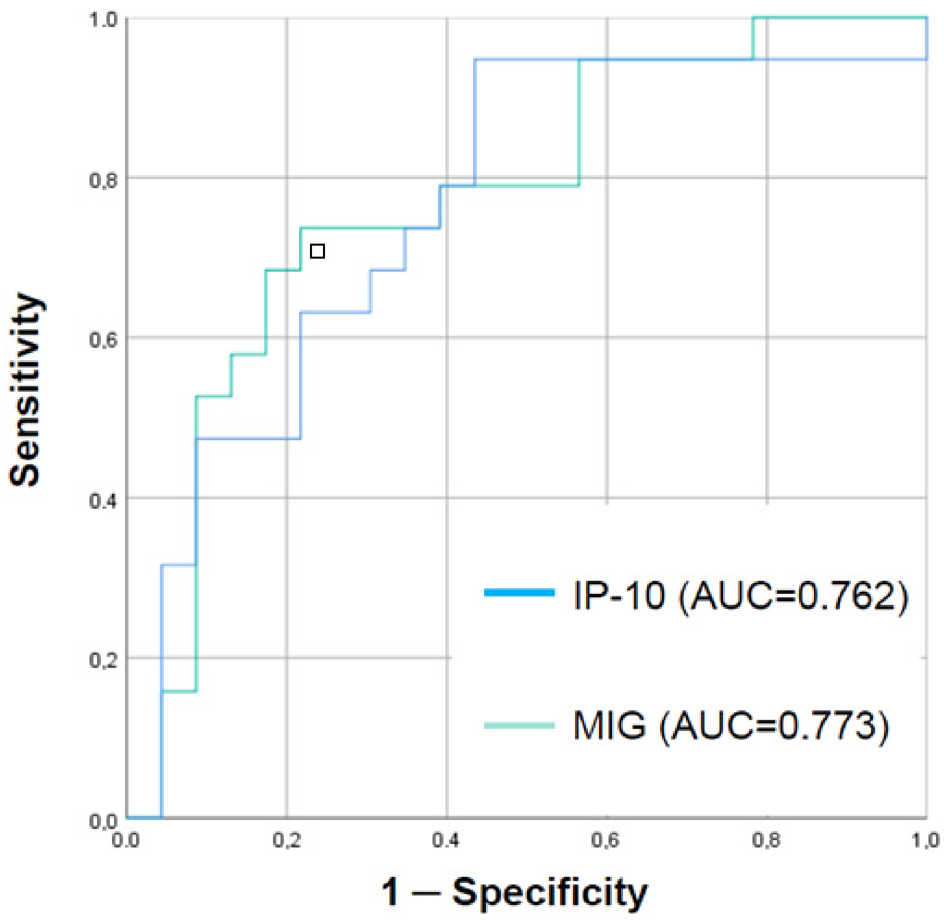

Figure S1. ROC curve for the prediction of a successful outcome of ART treatment AUC, area under the curve; ROC, receiver operating characteristics; ART, assisted reproductive technologies.

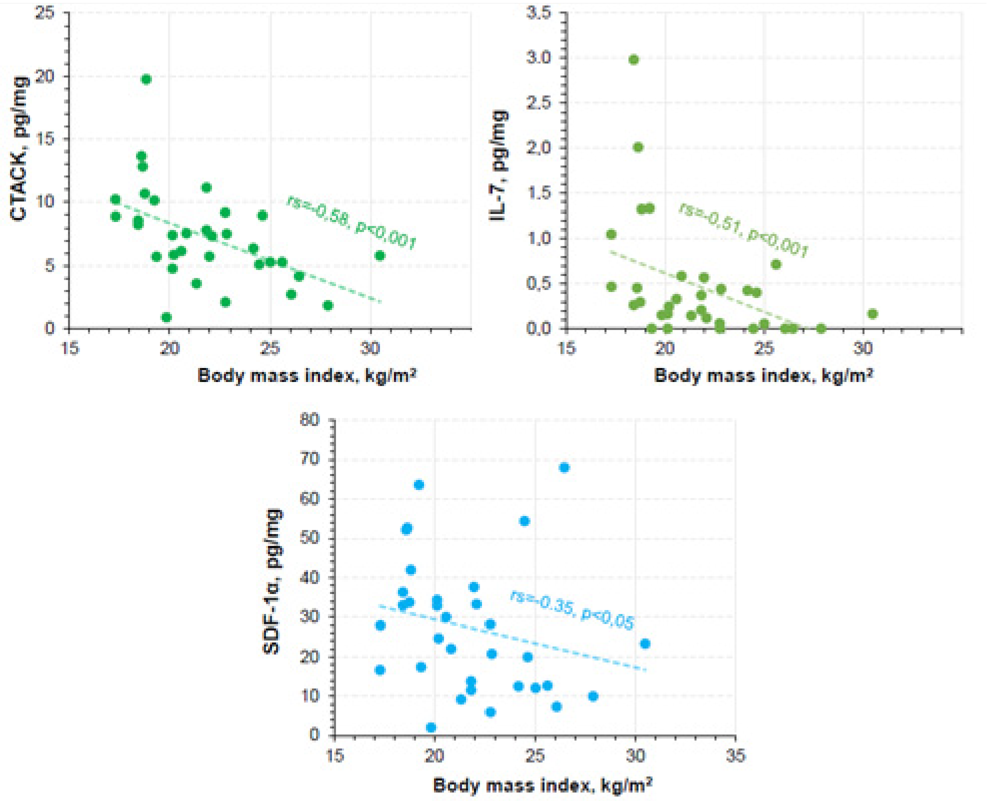

Figure S2. Scatterplots for the body-mass indexes and levels of immune mediators. Data are demonstrated only for immune mediators with significant correlations ($p < 0.05$). Data presented as pg of cytokine per mg of total protein in menstrual supernatant. Rs, Spearman's rank correlation coefficient.

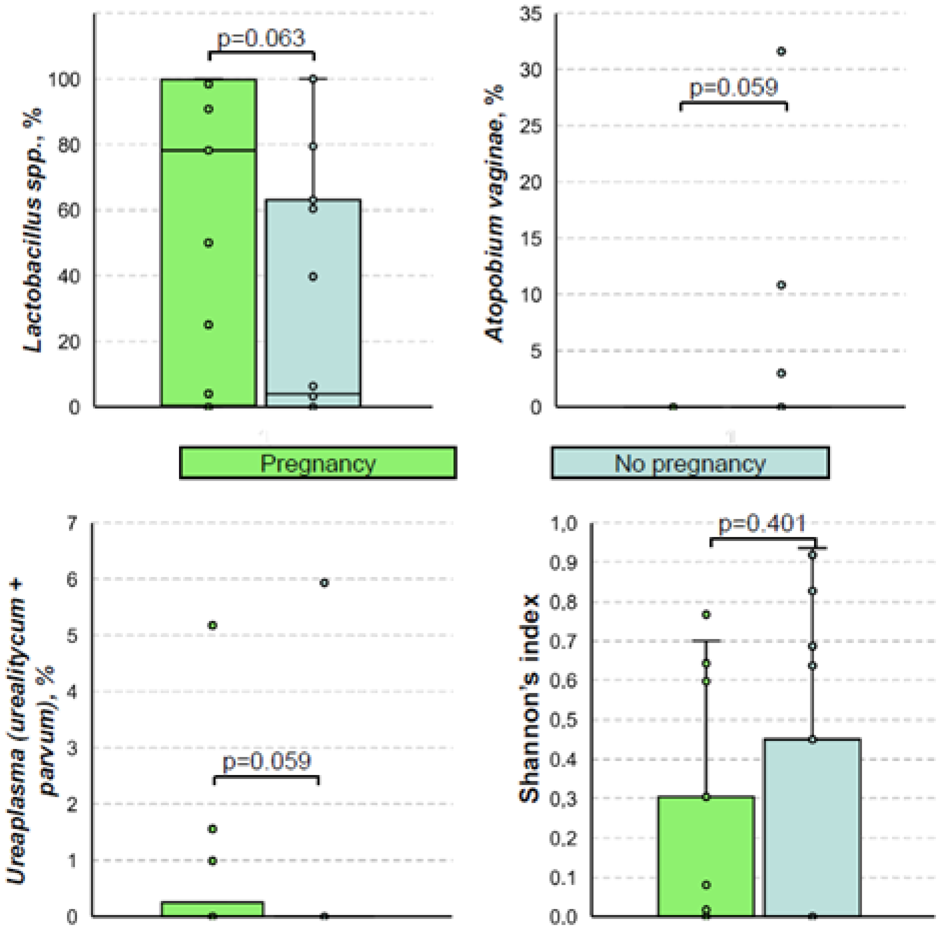

Figure S3. Boxplots for the comparison of abundances of microbial taxa and α-diversities. Data are demonstrated only for taxa with near-significant p-values. p-values were calculated using the Mann–Whitney U test. Data presented as abundance (% of total bacterial load measured via amplification of conservative prokaryotic DNA sequence). Shannon's α-diversity indexes are calculated based on the analyzed microbiota (mostly bacterial vaginosis-associated microorganisms and do not represent the real microbiological structure of the menstrual sediment).

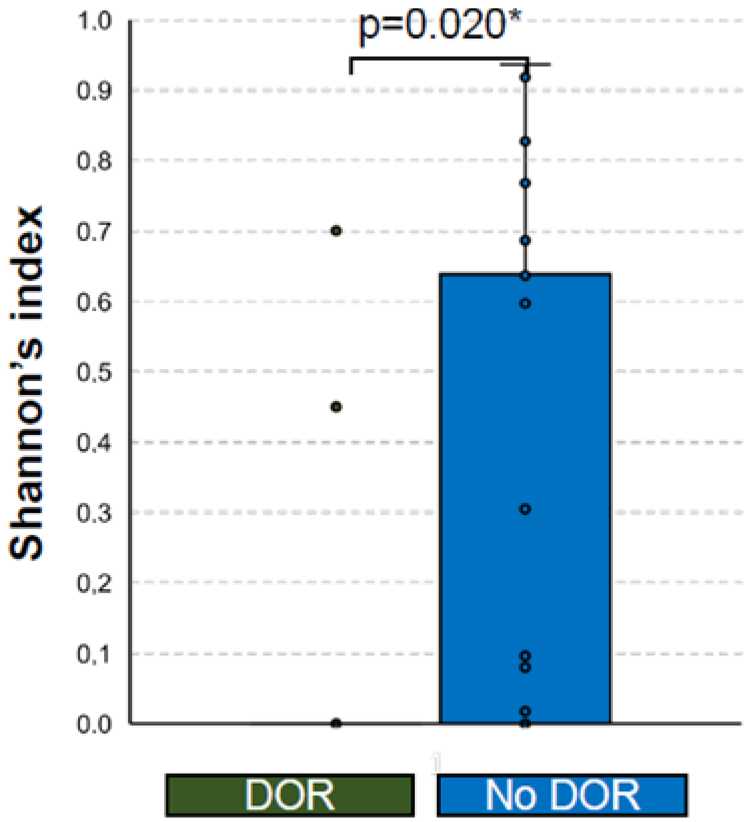

Figure S4. Boxplots for the comparison of α-diversities based on the presence of diminished ovarian reserve. DOR, diminished ovarian reserve. Data are demonstrated only for parameters with significant p-values. p-values were calculated using the Mann–Whitney U test. Shannon's α-diversity indexes were calculated based on the analyzed microbiota (mostly bacterial vaginosis-associated microorganisms that do not represent the real microbiological structure of the menstrual sediment). *, p-value significant after the Benjamini–Hochberg adjustment for multiple comparisons.

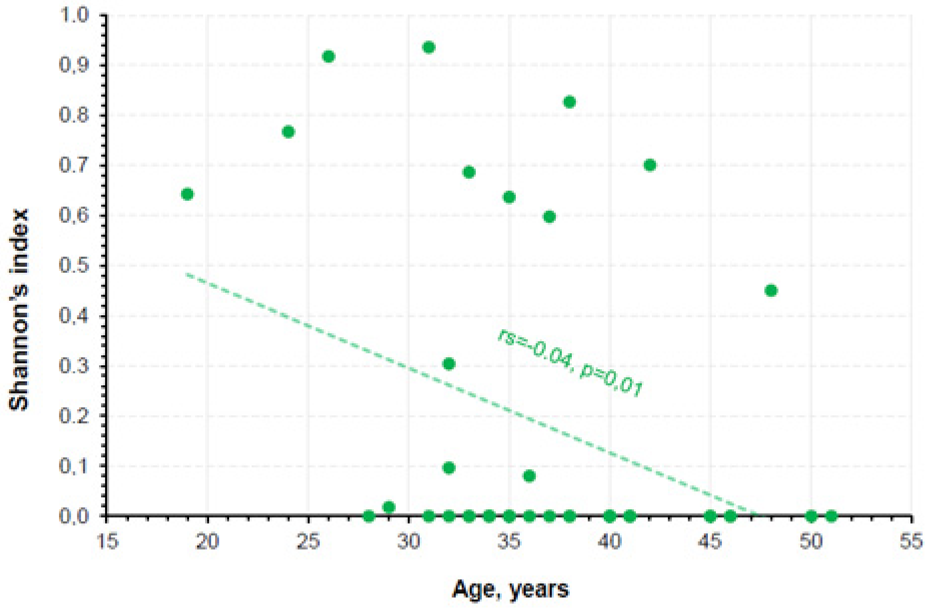

Figure S5. Scatterplot for the microbial α-diversities and age of study participants. Data are demonstrated only for significant correlations ($p < 0.05$). Shannon's α-diversity indexes are calculated based on the analyzed microbiota (mostly bacterial vaginosis-associated microorganisms and do not represent the real microbiological structure of the menstrual sediment). Rs, Spearman's rank correlation coefficient.

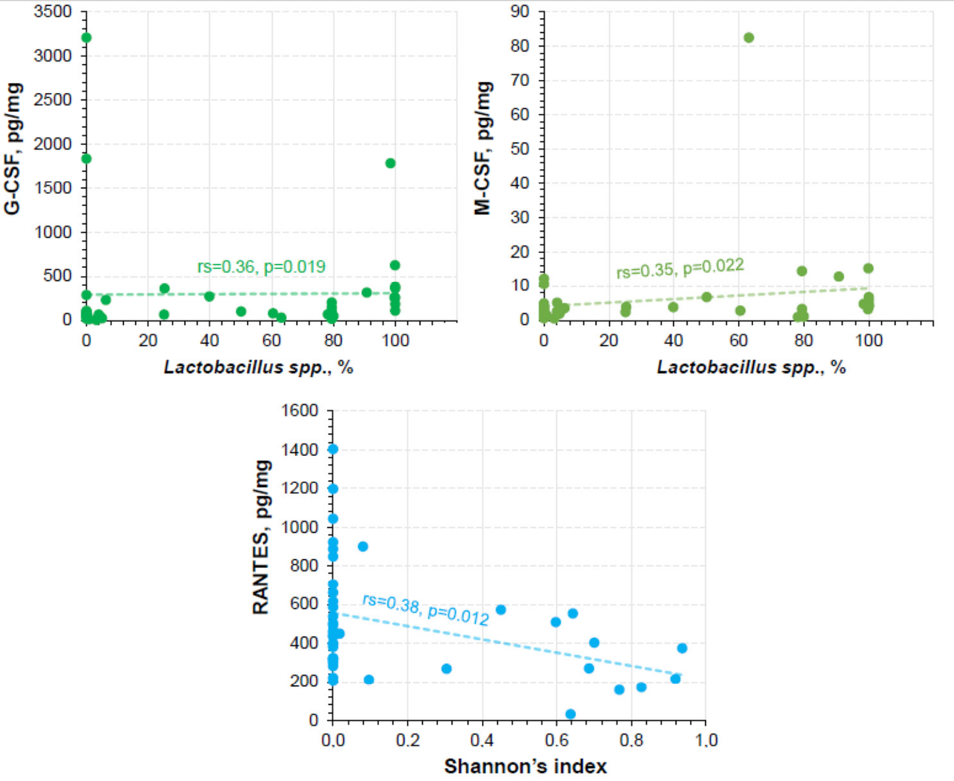

Figure S6. Scatterplots for the abundances of *Lactobacillus* spp., α-diversities, and levels of immune mediators. Data are demonstrated only for immune mediators with significant correlations ($p <$ 0,05). Data presented as abundance (% of total bacterial load measured via amplification of conservative prokaryotic DNA sequence) and pg of cytokine per mg of total protein in menstrual supernatant. Shannon's α-diversity indexes are calculated based on the analyzed microbiota (mostly bacterial vaginosis-associated microorganisms and do not represent the real microbiological structure of the menstrual sediment). Rs, Spearman's rank correlation coefficient.
