# Supplementary material for: Microbiological and Cytokine Profiling of Menstrual Blood for the Assessment of Endometrial Receptivity: A Pilot Study
Source: Biomedicines. 2023 Apr 26;11(5):1284. doi: 10.3390/biomedicines11051284 (PMC10215904; doi:10.3390/biomedicines11051284)
Supplement: Supplementary file 1 [file biomedicines-11-01284-s001.zip › biomedicines-2288794-Tables S2-S3.pdf]

Table S2. Complete results of cytokine analysis.

Immune Mediator	Pregnancy Group (n = 19)	No Pregnancy Group (n = 23)	p-value	Total Cohort (n = 42)
CTACK	6.155 [5.28; 10.369]	7.351 [4.46; 8.978]	0.850	7.020 [5.022; 9.036]
Eotaxin	1.393 [0.918; 2.013]	1.211 [0.69; 1.52]	0.240	1.277 [0.816; 1.693]
Basic FGF	65.353 [18.497; 105.04]	28.84 [9.691; 78.98]	0.230	31.254 [17.711; 81.384]
G-CSF	107.519 [65.197; 360.407]	65.634 [33.137; 184.054]	0.029	93.103 [49.708; 261.326]
GM-CSF	0.683 [0.482; 1.597]	0.719 [0.367; 2.013]	0.830	0.701 [0.372; 1.701]
GRO-alfa	313.532 [120.206; 817.382]	104.624 [54.064; 660.365]	0.027	205.284 [71.917; 664.030]
HGF	308.767 [170.272; 694.22]	277.272 [77.371; 477.282]	0.356	293.0195 [142.345; 573.614]
IFN-alfa2	0.166 [0; 0.532]	0.255 [0; 0.545]	0.969	0.185 [0; 0.535]
IFN-gamma	0.72 [0.212; 2.063]	0.317 [0.207; 0.53]	0.120	0.3845 [0.211; 0.916]
IL-1 alfa	14.879 [2.83; 30.492]	9.359 [5.479; 15.413]	0.426	10.066 [5.303; 26.320]
IL-1 beta	2.143 [1.005; 3.862]	1.279 [0.761; 4.043]	0.390	1.285 [0.834; 3.907]
IL-1ra	1378.713 [646.171; 1801.865]	911.501 [566.269; 1352.785]	0.161	1041.721 [608.461; 1714.641]
IL-2	0.464 [0.301; 0.732]	0.366 [0.187; 0.737]	0.426	0.3975 [0.281; 0.733]
IL-2R alfa	6.111 [4.168; 9.848]	4.397 [2.674; 6.911]	0.093	4.698 [2.964; 7.575]
IL-3	0.134 [0.078; 0.213]	0.088 [0.033; 0.219]	0.433	0.124 [0.066; 0.214]
IL-4	0.267 [0.148; 0.389]	0.209 [0.128; 0.246]	0.053	0.213 [0.128; 0.306]
IL-5	5.943 [0; 23.153]	4.187 [0; 10.822]	0.263	4.327 [0; 13.453]
IL-6	62.848 [28.498; 233.212]	36.413 [21.085; 61.874]	0.031	42.592 [24.191; 138.207]
IL-7	0.294 [0; 0.713]	0.206 [0.059; 0.404]	0.493	0.254 [0.057; 0.456]
IL-8	520.831 [285.648; 1376.679]	342.185 [179.236; 550.07]	0.060	404.328 [264.036; 812.608]
IL-9	31.385 [10.563; 40.98]	13.786 [8.472; 24.754]	0.037	15.364 [9.773; 35.669]
IL-10	1.357 [1.042; 2.53]	0.954 [0.441; 1.602]	0.073	1.177 [0.599; 2.116]
IL-12 (p70)	0.165 [0.083; 0.276]	0.095 [0.049; 0.201]	0.128	0.123 [0.056; 0.251]
IL-12 (p40)	23.319 [10.863; 44.754]	15.476 [6.307; 29.956]	0.245	20.385 [10.751; 33.999]
IL-13	0.046 [0.014; 0.116]	0.029 [0.02; 0.055]	0.604	0.037 [0.017; 0.065]
IL-15	27.897 [13.482; 53.112]	16.602 [9.502; 25.986]	0.079	19.826 [12.687; 37.928]
IL-16	34.099 [20.335; 80.356]	26.037 [10.457; 43.041]	0.071	27.625 [15.7193; 51.453]
IL-17	1.107 [0.486; 1.469]	0.969 [0.654; 1.653]	0.649	1.064 [0.506; 1.634]
IL-18	4.287 [3.29; 5.338]	3.895 [1.688; 5.383]	0.331	4.179 [1.929; 5.349]
IP-10	39.098 [21.667; 114.229]	17.695 [11.187; 33.574]	0.004*	26.0065 [15.869; 46.712]
LIF	40.689 [16.101; 49.141]	26.307 [16.277; 33.505]	0.109	27.7535 [16.233; 44.886]
MCP-1	9.302 [4.164; 42.019]	3.851 [2.562; 10.563]	0.027	6.623 [3.417; 15.210]
MCP-3	0.069 [0; 0.18]	0.078 [0; 0.158]	0.837	0.0725 [0; 0.170]
M-CSF	4.782 [3.252; 6.775]	2.57 [1.352; 3.526]	0.012*	3.317 [1.586; 4.971]
MIF	520.779 [380.784; 1655.352]	564.719 [215.705; 978.885]	0.261	542.749 [241.071; 1193.262]
MIG	15.428 [7.753; 31.222]	7.168 [4.318; 10.97]	0.003*	10.345 [4.919; 19.360]
MIP-1 alfa	2.175 [1.093; 4.191]	1.387 [1.153; 2.855]	0.337	1.95 [1.138; 3.318]
MIP-1 beta	41.133 [24.824; 55.916]	26.217 [12.237; 74.974]	0.503	39.852 [16.787; 59.492]
beta-NGF	1.146 [0.725; 2.169]	1.005 [0.539; 2.081]	0.640	1.103 [0.680; 2.103]
PDGF-BB	24.33 [17.019; 66.075]	36.107 [18.308; 66.783]	0.695	31.105 [17.986; 66.252]
RANTES	438.391 [317.695; 704.62]	435.46 [270.846; 572.891]	0.536	436.926 [294.749; 594.091]
SCF	3.102 [2.272; 5.966]	2.007 [1.886; 3.413]	0.018*	2.6485 [1.959; 3.804]
SCGF-beta	7045.489 [4375.593; 9077.084]	5419.363 [2869.97; 8252.371]	0.120	5845.911 [3313.462; 8297.142]
SDF-1 alfa	29.952 [20.691; 41.918]	19.93 [11.542; 32.89]	0.039	23.0875 [12.614; 33.897]
TNF-alfa	7.772 [4.667; 13.895]	6.14 [3.253; 7.022]	0.083	6.252 [4.033; 9.678]
TNF-beta	49.755 [16.1; 58.076]	21.796 [12.253; 36.455]	0.031	25.137 [14.621; 53.560]

TRAIL	8.769 [5.785; 17.507]	4.7 [2.645; 8.433]	0.010*	6.63 [3.111; 11.442]
VEGF	21.837 [5.359; 28.543]	8.522 [5.093; 15.753]	0.067	12.960 [5.293; 22.714]

Data presented as pg of analyte per mg of total protein. *p*-values were calculated using Mann-Whitney U test and are presented for the comparison of groups "pregnancy" vs. "no pregnancy". *, *p*-values significant after the Benjamini-Hochberg adjustment for multiple comparisons.

Table S3. Complete results of correlation analysis for hemoglobin and all the studied immune mediators.

Immune Mediator	Spearman's Rank Correlation Coefficient for Hemoglobin	*p*-Value
CTACK	0.533	0.001*
Eotaxin	0.378	0.014*
Basic FGF	0.181	0.252
G-CSF	0.045	0.775
GM-CSF	0.311	0.045*
GRO-alfa	0.005	0.975
HGF	0.271	0.082
IFN-alfa2	0.169	0.284
IFN-gamma	−0.029	0.854
IL-1 alfa	0.258	0.099
IL-1 beta	0.262	0.094
IL-1ra	0.281	0.071
IL-2	0.273	0.080
IL-2R alfa	0.226	0.150
IL-3	0.259	0.098
IL-4	0.323	0.037*
IL-5	−0.040	0.803
IL-6	−0.048	0.761
IL-7	0.286	0.066
IL-8	0.126	0.427
IL-9	0.429	0.005*
IL-10	0.340	0.027*
IL-12 (p70)	0.187	0.235
IL-12 (p40)	0.297	0.056
IL-13	0.218	0.166
IL-15	0.244	0.120
IL-16	0.170	0.282
IL-17	0.237	0.130
IL-18	0.432	0.004*
IP-10	−0.103	0.516
LIF	0.133	0.400
MCP-1	−0.114	0.473
MCP-3	0.064	0.688
M-CSF	0.234	0.135
MIF	−0.006	0.970
MIG	0.126	0.428
MIP-1 alfa	0.271	0.083
MIP-1 beta	0.365	0.017*
beta-NGF	0.317	0.041*
PDGF-BB	0.616	0.001*
RANTES	0.651	0.001*

SCF	0.333	0.031*
SCGF-beta	0.243	0.121
SDF-1 alfa	0.454	0.003*
TNF-alfa	0.153	0.334
TNF-beta	0.452	0.003*
TRAIL	−0.139	0.381
VEGF	0.022	0.888

Correlation was determined for values not normalized by total protein levels. *. *p*-values less than 0.05.
